# Capacity, responsibility, and motivation: a critical qualitative evaluation of patient and practitioner views about barriers to self-management in people with multimorbidity

**DOI:** 10.1186/s12913-014-0536-y

**Published:** 2014-10-31

**Authors:** Peter A Coventry, Louise Fisher, Cassandra Kenning, Penny Bee, Peter Bower

**Affiliations:** NIHR Collaboration for Leadership in Applied Health Research and Care Greater Manchester and Manchester Academic Health Science Centre, University of Manchester, Oxford Road, Manchester, M13 9PL UK; NIHR School for Primary Care Research and Manchester Academic Health Science Centre, University of Manchester, Manchester, M13 9PL UK; School of Nursing, Midwifery and Social Work, University of Manchester, Manchester, M13 9PL UK

## Abstract

**Background:**

Primary care is increasingly focussed on the care of people with two or more long-term conditions (*multimorbidity*). The UK Department of Health strategy for long term conditions is to use self-management support for the majority of patients but there is evidence of limited engagement among primary care professionals and patients with multimorbidity. Furthermore, multimorbidity is more common in areas of socioeconomic deprivation but deprivation may act as a barrier to patient engagement in self-management practices.

**Background:**

Effective self-management is considered critical to meet the needs of people living with long term conditions but achieving this is a significant challenge in patients with multimorbidity. This study aimed to explore patient and practitioner views on factors influencing engagement in self-management in the context of multimorbidity.

**Methods:**

A qualitative study using individual semi-structured interviews with 20 patients and 20 practitioners drawn from four general practices in Greater Manchester situated in areas of high and low social deprivation.

Patients were purposively sampled on socioeconomic deprivation (defined by Index of Multiple Deprivation (IMD) score), number and type of long term conditions (2 or more of: coronary heart disease, diabetes mellitus, osteoarthritis, chronic obstructive pulmonary disease and depression), age and gender. Practitioners were sampled by deprivation status of the practice area; role (i.e. salaried GP, GP principal, practice nurse); and number of years’ experience. Interviews were audio-recorded and transcribed verbatim. Analysis used a thematic approach based on Framework.

**Results:**

Three main factors were identified as influencing patient engagement in self-management: *capacity* (access and availability of socio-economic resources and time; knowledge; and emotional and physical energy), *responsibility* (the degree to which patients and practitioners agreed about the division of labour about chronic disease management, including self-management) and *motivation* (willingness to take-up types of self-management practices). Socioeconomic deprivation negatively impacted on all three factors. Motivation was especially reduced in the presence of mental and physical multimorbidity.

**Conclusion:**

Full engagement in self-management practices in multimorbidity was only present where patients’ articulated a sense of capacity, responsibility, and motivation. Patient ‘know-how’ or interpretive capacity to self-manage multimorbidity is potentially an important precursor to responsibility and motivation, and might be a critical target for intervention. However, individual and social resources are needed to generate capacity, responsibility, and motivation for self-management, pointing to a balanced role for health services and wider enabling networks.

**Electronic supplementary material:**

The online version of this article (doi:10.1186/s12913-014-0536-y) contains supplementary material, which is available to authorized users.

## Background

Primary care is increasingly focussed on the care of people with long term conditions – many of whom live with two or more such conditions, a status known as *multimorbidity* [1]. While it could be argued that depression is not a single entity it is a syndrome with recognisable symptoms that follow a relapsing and remitting course, lending depression many of the features of a long term condition [2]. In this sense multimorbidity as a concept can include any combination of physical and mental health conditions. The prevalence of multimorbidity varies according to definition and population but was recently estimated to affect 16% of all patients in England and 44% of those aged 75 or more [3]. However, multimorbidity is not a problem confined to older adults. In socioeconomically deprived areas multimorbidity occurs 10 to 15 years earlier and more commonly includes mental health disorders [4]. So called physical and mental multimorbidity is associated with greater decrements in health than other disease combinations [5], and increases the risk of unplanned hospital admissions [6,7]. The presence of physical and mental multimorbidity also significantly impacts the cost of health care. International estimates suggest that health care costs increase by at least 45 per cent for each person with a chronic physical disease and a co-morbid mental health problem [8].

Effective self-management is considered critical to meet the needs of people living with long-term conditions. In the UK, self-management has been defined as “the care taken by individuals towards their own health and well being: it comprises the actions they take to lead a healthy lifestyle; to meet their social, emotional and psychological needs; to care for their long-term condition; and to prevent further illness or accidents” [9]. Here, the emphasis on lifestyle is predicated on a belief that by engaging in healthy behaviours patients can limit further disease progression, and avoid the need for more intensive level of support and thus reduce healthcare utilisation and cost. The means by which the health service can support patients with long term conditions to engage in self-management include appropriate and accessible advice, health education, self-care skills training and increasingly self-monitoring via tele-health technologies [9,10].

Encouraging self-management in primary care is difficult [11], and due to the accumulative demands of two or more long term conditions, the presence of multimorbidity may be a further barrier to patient engagement in self-management. Patients with multimorbidity may have less energy, time, and motivation to devote to complex self-management activities [12]. Owing to the complexity of information about treatment regimens even the most motivated and well informed patients with multimorbidity may struggle to make the right self-management decisions [13]. Furthermore, because older patients with multimorbidity may be at higher risk of normal age-related deficits in cognitive functioning, their ability to engage successfully in self-management tasks might be significantly impaired. Additionally, depression may complicate self and medical management of long term conditions [14,15]. Self-management support in the context of multimorbidity may also tax the clinical skills of professionals thereby limiting their ability to support complex patients to self-manage. This may be especially so in socio-economically deprived areas where historically the provision of health care has rarely met the needs of the most ill and disadvantaged [16].

While there is some evidence that patients with multimorbidity may benefit the most from self-management support programmes [17,18], most have been designed for people with single long term conditions and as such may have less relevance for people with multimorbidity. Even patient level behavioural interventions to support self-management in multimorbidity have met with only modest success, further highlighting the limited scope of evidence about how best to manage multimorbidity [19].

If self-management is really to address the challenge of improving the health of patients with multimorbidity and reduce health service utilisation, health services need to understand how best to engage and support patients and practitioners to introduce improvements in self-management. We therefore conducted a qualitative study to explore patient and professional perspectives on the factors that facilitate and hinder patient engagement within self-management practices in the context of multimorbidity.

## Methods

### Setting and recruitment

This qualitative study was nested in a larger quantitative study designed to explore predictors of self-management behaviour in patients with multimorbidity. The cohort study surveyed 1500 patients from four general practices in Greater Manchester. Patients were selected on the presence of two or more of five exemplar conditions (coronary heart disease, diabetes mellitus, osteoarthritis, chronic obstructive pulmonary disease and depression). These conditions were chosen on the basis that they have varied symptomatology and present patients and clinicians different management challenges. 516 participants (34%) responded to the invitation to complete the survey, of which in 222 (i.e. 43%) gave consent to be approached for interview. From this group 20 patients were purposively sampled on socioeconomic deprivation (defined by Index of Multiple Deprivation (IMD) score [20]), number and type of long term conditions, age and gender. Characteristics of the patient sample are shown in Table 1. Fifteen practitioners were initially recruited from the four practices participating in the quantitative study, and a further 5 were recruited from three other practices where the researchers had prior links. Due to difficulties with recruitment, practitioners were recruited using convenience sampling but attempts were still made to interview subjects varying in characteristics of interest such as: deprivation status of the practice area; role (i.e. salaried GP, GP principal, practice nurse); and number of years’ experience. In total 16 GPs and 4 Practice Nurses consented to interview; characteristics of the practitioner sample are shown in Table 2.

**Table 1:** **Patient characteristics**

**Patient ID**	**Age**	**Practice number**	**IMD score**	**Conditions**
P1	68	1	11.59	Osteoarthritis (OA)
				Coronary heart disease (CHD)
				Depression
P2	76	1	33.08	COPD
				CHD
				Depression
P3	57	4	5.39	OA
				CHD
P4	58	2	46.55	CHD
				Depression
P5	58	3	19.96	Diabetes
				CHD
P6	88	3	9.71	COPD
				OA
				CHD
P7	54	3	19.96	Asthma
				Diabetes
				OA
				Depression
P8	67	3	15.68	Diabetes
				CHD
P9	76	1	26.71	Diabetes
				COPD
				CHD
				Depression
P10	68	1	30.12	OA
				CHD
				Depression
P11	76	4	11.15	OA
				CHD
P12	57	1	43.81	Diabetes
				OA
P13	77	4	10.92	OA
				CHD
P14	65	4	53.17	Diabetes
				COPD
				OA
				Depression
P15	52	1	27.21	Diabetes
				OA
				Depression
P16	58	2	66.68	Asthma
				Diabetes
P17	63	4	9.03	Diabetes
				Depression
P18	76	4	16.17	COPD
				CHD
P19	66	2	25.17	COPD
				CHD
P20	58	3	6.73	Diabetes
				Depression

**Table 2:** **Practitioner characteristics**

**Practitioner ID**	**Practice number**	**IMD score**	**Number of years qualified**	**Role (e.g. salaried/partner etc)**
DR 1	1	44.76	30	GP Partner
DR 2	3	19.96	21	GP Partner
DR 3	1	44.76	12	Salaried GP
DR 4	1	44.76	17	GP Partner
DR 5	1	44.76	8	GP Partner
DR 6	2	25.17	30	GP Partner
DR 7	5	7.1	16	GP Partner
DR 8	4	11.15	11	Salaried GP
DR 9	6	6.53	23	GP Partner
DR 10	1	44.76	5	Trainee GP
DR 11	4	11.15	5	Trainee GP
DR 12	4	11.15	20	GP Partner
DR 13	5	7.1	16	GP Partner
DR 14	4	11.15	36	GP Partner
DR 15	5	7.1	23	GP Partner
DR 16	7	50.74	5	Trainee GP
PN 1	1	44.76	22	Practice Nurse
PN 2	4	11.15	27	Practice Nurse
PN 3	1	44.76	5	Healthcare assistant
PN 4	2	25.17	25	Practice Nurse

While Framework analysis does not explicitly provide guidance about sample size our criterion based sampling approach meant that we aimed to include at least 5 patients per criterion: age, gender, combination of illnesses, and level of deprivation. Based on previous experience of using semi-structured interviews in applied health research settings [21] we anticipated that data saturation in the patient and practitioner data set would be achieved after a minimum of 20 purposively sampled interviews. We aimed to interview at least 5 cases per sampling criterion for both the patient and practitioner samples.

### Data collection

Individual semi-structured interviews were conducted with patients and practitioners by one of two authors (LF and CK). A topic guide covering the main themes was used (see Additional files 1 and 2). Patient interviews were conducted in their own homes and practitioners were interviewed at locations according to their preference. The average length of interview was 38 minutes (range 10-72). All interviews were digitally recorded with consent and transcribed verbatim.

### Data analysis

Interviews were analysed to explore a priori and emergent themes using an approach informed by Framework [22]. Five key steps were followed: 1) familiarisation – the transcripts were read thoroughly by all researchers to identify key themes; 2) a preliminary thematic framework was constructed using the interview schedules to structure the early themes. 3) indexing – themes and emerging sub themes were labelled and indexed; 4) charting – each framework was converted into a series of thematic charts; 5) mapping and interpretation – the key characteristics across all the data were mapped and interpreted. Disconfirming evidence and deviant cases were sought throughout the analysis [23].

Analysis was carried out by four researchers from different backgrounds (general practice, health services research and health psychology) to increase trustworthiness of analysis [24]. Each transcript was analysed individually and then in groups, with the healthcare professional transcripts analysed separately from the patient transcripts but with comparisons made across data sets. In doing so this qualitative study drew on the concept of investigator triangulation by sharing data collection and data analysis between researchers drawn from different disciplinary backgrounds, again increasing trustworthiness of the analysis [25].

### Ethics statement

Ethical approval was granted by Greater Manchester North Ethics Committee on 12/09/2011 (ref: 11/NW/0563). Approvals included permissions from participants to use their data in an anonymised format: patient names were removed but clinical and demographic data are included to characterise the type of patients interviewed; names of professionals were removed but demographic data and information about service length are included to facilitate comparisons along gender and years of experience.

This study was conducted in accordance with the RATS guidelines on qualitative research (http://www.biomedcentral.com/ifora/rats).

## Results

Core to all the interviews were three themes that captured patients’ and professionals’ views as to how successful self-management in multimorbidity hinged on the interplay and interdependence between contextual factors related to patients’ material and social resources, physiological characteristics associated with patients’ health, and patients’ attitude to health care. Data saturation was achieved in the patient data set; because only four practice nurses were interviewed it is likely that the practitioner data set was dominated by themes generated by the GP participants. These themes were:

1. Capacity: which included capacity external to the patient (access to social and economic infrastructure and time to support self-management activities), their capacity in terms of know-how and confidence to accomplish complex self-management practices; and physical and emotional capacity to focus on self-management.
2. Responsibility: which centred on patients’ and practitioners’ attitudes about the division of labour associated with self-management and medical management in multimorbidity, and how these attitudes were partly contingent on capacity.
3. Motivation: which drew on understandings that successful self-management was partly contingent on patients’ belief and expectation that self-management would improve their health, and how low mood can negatively influence patients’ capacity and sense of responsibility for self-management.

The extent to which these themes were perceived to be significant was influenced by the degree to which practitioners and patients encountered socio-economic deprivation, highlighting the importance of deprivation in engaging people with multimorbidity in self-management.

### Capacity

When discussing barriers to self-management in multimorbidity, practitioner narratives initially focused on the fact that many patient with multiple health problems often expended a great deal of energy and time coping with day-to-day routines associated with living with illness, leaving them with little spare capacity to devote to more complex self-management tasks:

“*I speculate that with several conditions people are too busy just trying to survive. [Their day is spent] getting up in the morning out of bed (if they can), having a plateful, almost a meal full, of tablets every day, and just about coping on the edge of everyday life.”* DR 1 (30 yrs qualified: GP principal, deprived area)

Physiological barriers, generated from competing physical conditions, were also at the forefront of practitioners’ minds when discussing the problems encountered by patients with multimorbidity. For example, practitioners knew of patients with combinations of illnesses or levels of physical incapacity that precluded self-management tasks that involved lifestyle changes such as exercise:

*“Somebody with diabetes… you encourage them to exercise, [but] maybe if they’ve got a respiratory condition, it stops them from doing that. So sometimes your advice conflicts, you know, when you’ve got multiple problems.”* PN 2 (27 yrs qualified: Practice Nurse)

In contrast to these professional concerns, which centred on balancing advice about risks and benefits of self-management, some patients had greater interpretive capacity (i.e. know-how or tacit knowledge) to spot opportunities to maximise the benefits of self-management for all their health problems:

*“in truth, a lot of the things are similar for both… exercise is good for my heart and it’s good for my diabetes, and paced exercise is good for the late effects of polio, improving your diet; it’s good for all conditions, really.”* Patient 5 (58 yrs: diabetes, CHD)

Structural factors, such as access to transport or financial resources, were considered by patients as providing important and tangible ingredients in generating capacity to self-manage. Additionally, many patients spoke about how their capacity to cope with their multiple health problems was sustained through the perceived social and emotional support provided by their family (who often acted as informal carers), friends, and sometimes community and religious groups. Here, perceived emotional support provided a solid base from which patients could cope with and self-manage multimorbidity:

*“the only thing that’s helped me through this [multiple illness] is being part of a church, and some good friends in the church, who have been kind, and helpful over the years, and supportive.”* Patient 7 (54 yrs: asthma, diabetes, OA, depression)

Equally, practitioners also placed a high value on patients’ being able to mobilise a network of support to help them in managing their health problems. Social isolation was seen to reduce patients’ capacity to engage in self-management activities outside the home, often because they had no family nearby or poor access to social networks that might support them to learn about self-management:

*“I think if there’s social isolation that can be quite a big problem. So social isolation where they can't get out, where they can’t use ordinary channels of communication… They’ve no relatives, no friends, and they’re just stuck at home.”* DR 14 (36 yrs qualified: GP principal)

Practitioners suggested that poor access to material resources further eroded patients’ capacity to engage in self-management tasks. This was especially true for patients who lived in more socio-economically deprived areas. Patients who lived in more deprived areas were not only less likely to have fewer financial resources but also had limited access to public or private transport, leading to poorer up take of self-management options such as support groups:

*“Obviously, with low socioeconomic background.....you may not have the facilities…to do certain things; self care depends, in some part on…, things like access to telephones, access to internet, being able to go to some of these classes by public transport, and…some patients may not have that*.” DR 10 (5 yrs qualified: GP trainee, deprived area)

Quite apart from practitioner concerns about how deprivation impacts negatively on structural capacity for self-management some patients from deprived backgrounds articulated how lack of financial resources dented their emotional capacity to invest in learning about and doing self-management. For these patients, especially those who relied on benefit payments, daily anxieties about money meant that their time and energy was spent on making ends meet, not seeking out opportunities to enhance self-management practices:

*“They’re only giving me £14 a week to live on. Out of that £14 I’ve got to pay £17 a week for water and heating. That’s another thing that does your head in because how are you supposed to live…? It’s playing on your mind all the time.”* Patient 4 (58 yrs: CHD, depression, deprived area)

By comparison patients with greater financial resources acknowledged that their relative affluence enhanced their emotional as well as structural capacity to devote to caring for themselves. For example, one patient who had previously had a heart attack and had continued to work in a stressful job reflected on how an opportunity to take up a lucrative early retirement had enhanced his capacity to control his heart disease:

*“I was a sales manager and I was driving 1,000 miles a week and my doctor was always saying ‘it’s killing you…you can’t go on to driving 1,000 miles a week and all the pressures of sales’, so in 2000 I was offered a redundancy package, I was only 58 at the time and, but we [sales managers] retire at 60, so I was lucky that I got a good pension, a redundancy package and I finished work and at that point I felt almost in total control, whereas before it [the heart disease] was at the influence of other sorts of things.”* Patient 8 (67 yrs: diabetes, CHD)

### Responsibility

Although both practitioners and patients identified capacity as a necessary feature of self-management it is not sufficient to ensure patient engagement with self-management. In addition to capacity patients and practitioners identified responsibility as a key ingredient in successful self-management. This theme revolved around practitioners’ and patients’ discourse about their respective roles to support self-management.. In this sense responsibility stemmed from patients’ capacity to act out self-management tasks and relates in part to established notions of self-efficacy (i.e. confidence to produce a desired outcome) [26]. Because much self-management activity takes place outside of formal health care and is patient led, practitioners believed that patients should take charge of all tasks associated with healthy lifestyles and medicines management:

*“if the patient was at home, maybe, eating unhealthy food, not taking their medications and there’s little [I can do]…I can’t go in and, you know, do that for them, so, I think, people need to take more responsibility for their own conditions.”* DR 12 (20 yrs qualified: GP principal)

However, practitioners were not inclined to believe that patients with multimorbidity should be less or more responsible for their health than patients with single long term conditions. Practitioners noted that all patients, regardless of the number of illnesses, have a responsibility to maintain their health, but the degree to which this was true might vary dependent on patients’ capacity, especially their interpretive capacity to process and understand complex advice about self-management tasks:

*“I think the responsibility is the same, because as long as the patient can weigh up the information that you give them, it doesn’t really make any difference if it’s one illness or two illnesses.”* DR 4 (female, 17 yrs qualified: GP principal, deprived area)

The extent to which responsibility was contingent on capacity was also articulated through the experiences of practitioners who worked in deprived areas. In these areas practitioners recognised that patients displayed lower levels of responsibility towards self-management, entrusting their care instead to formal health care providers. Importantly, in making these observations, practitioners did not invoke a moral discourse about deprivation and dependency, but rather suggested that patients from deprived areas had less capacity to self-manage and were thus more reliant on their support than patients from less deprived areas:

*“I would probably say the patients here with chronic conditions probably expect doctors to fix it rather than taking care of themselves. They’re very dependent on GPs and doctors, I don’t know why. Maybe again, because it’s a deprived area.”* DR 3 (12 yrs qualified: salaried GP, deprived area)

Patients’ views on responsibility for health care appeared to confirm practitioners’ accounts. Patients from more deprived areas commonly relied on a narrative that suggested that responsibility for health lay firmly with medical professionals. However, in doing so, some patients also alluded to the notion that responsibility for self-management might also equate to, or at least include, compliance with health professionals’ advice. Here, responsibility for self-management becomes less about acting out behavioural tasks and more about complying with instructions about medical management of illness:

*“I think it’s the doctor’s concern, not mine. I mean I’ll do everything the doctor tells me.”* Patient 19 (66 yrs: COPD, CHD, deprived area)

Patients living in less deprived areas were less ambiguous about their role in self-management, expressing a belief that they, rather than medical professionals, should take ownership of their health problems. Here, looking after themselves might include adopting healthy behaviours but, echoing the sentiments of patients from more deprived areas, they also alluded to responsibility as compliance with medical management of illness:

*“I think to look after me is my responsibility, because if I said, ‘Oh, I’m not bothered because of bad weather, so I’m not ringing for some medication’ or something like that, so it is my fault, not theirs [if I don’t get my medications].”* Patient 6 (88 yrs, COPD, OA, CHD)

### Motivation

Thus far we have described how practitioners and patients characterised self-management in multimorbidity as being contingent first on capacity and secondly on responsibility. However, the last set of data in the previous section hinted at how full engagement in self-management is also only likely to be possible in the presence of patient motivation to overcome barriers and to extend their self-management style to include all tasks, not just medicines taking. Chief among barriers identified by practitioners was the common occurrence of depression in patients with multiple health problems. Because ‘low mood…goes along with poor motivation and certainly will affect how they manage their condition’ (DR 5) practitioners prioritised the management of depression in order to facilitate discussions with patients about adopting healthier lifestyles:

*“if you have someone who has got COPD and is depressed then obviously I think tackling depression will be my first priority because that will help me to motivate the patient, maybe to adapt their diet, or stop [them] smoking rather than just ignore it or not tackle it.”* DR 3 (12 yrs qualified: salaried GP, deprived area)

Patients concurred with practitioners that depression was an obstacle to self-management. Even where patients had expressed a commitment to adopt healthier lifestyles they recognised that depression could confound their desire to enact such self-management plans:

*“as you get older like I want to be fitter in myself and then… these little conditions, they stop you doing things, and then your motivation, if you’re feeling down and you’re depressed, then your motivation’s not there.”* Patient 15 (52 yrs: OA, diabetes, depression, deprived area)

In addition to the problem of motivating patients to make behavioural changes practitioners also highlighted that some patients, even in the absence of depression, were unlikely to feel motivated to attend support groups for people with multiple health problems. In accounting for why patients were typically dismissive of self-management groups practitioners pointed to the fact that most patients with multimorbidity will not have encountered such a group before and hold no expectation that they can improve their health:

*“this extra thing* [a self-management support programme] *is like an added hassle that they don’t perceive as being of any benefit. I see it that it probably would be beneficial and there’s no proof until they’ve been, and they don’t go because they don’t see it as a priority… It’s not as likely to work as a big red tablet.”* DR 1 (30 yrs qualified: GP principal, deprived area)

Self-management falls outside the traditional, and for some patients, the normatively circumscribed concept of health care, and as such, demands additional levels of motivation. Patients who did not expect that self-management support groups were likely to yield additional benefit reverted to a self-management style that was reliant on taking advice from their general practitioner:

*“it* [a self-management support programme] *doesn’t appeal to me quite honest – I feel whatever me doctors says seems to be sufficient, and we’re living with that.”* Patient 2 (76 yrs: COPD, CHD, depression, deprived area)

As well as patients’ low expectations and normative assumptions about self-management practitioners also pointed to socioeconomic deprivation as a factor that negatively impacted motivation among some patients. Echoing sentiments expressed earlier about patients’ emotional capacity practitioners declared that patients who were battling financial worries owing to unemployment felt much less empowered and thereby motivated to prioritise their health:

*“there’s other ones who don’t have much aspirations, who don’t work and they’re in chronic poor health and they feel there’s nothing they can do, they feel powerless, probably…[and] the thing is, they’ve got other things to worry about, maybe, paying their bills, poor housing… I think health must come way down the list for these people.”* DR 12 (20 yrs qualified: GP principal)

Practitioners working in deprived areas also noted that patients were heavily influenced by their environment in which poor health and indeed poor life expectancy was an accepted feature of life. In this sense patients from more deprived areas were socialised into expecting ill health and consequently felt less motivated to improve their health by adopting health protective behaviours:

*“sometimes people almost see it as normal, because they are surrounded by other people that are ill and neighbours that are ill and so I don’t think that necessarily they would look at themselves as being that unusual for the area.”* DR 4 (17 yrs qualified: GP principal, deprived area)

This perspective seemed to be borne out from patients’ narratives which revealed that patients from deprived areas were only motivated to carry on as they were rather than pushing themselves to take on new self-management roles:

*“I just plod along in the way I am. I seem to be coping alright, but as long as I behave myself and not try and do too much we are okay.”* Patient 2 (76 yrs: COPD, CHD, depression, deprived area)

By contrast, patients from less deprived areas (and thus with higher levels of capacity) shared a more positive outlook about their future heath and felt obliged to engage in health protective behaviours:

*“And instead of thinking, ‘Oh I’d love to go for a walk but I can’t,’ and sitting down and feeling grumpy about it, I’ll go off and swim…I know people in the pool now, where I exercise, so it’s about getting out of the house as well, and doing all of that.” Patient 3 (57 yrs: OA, CHD)*

## Discussion

### Main findings and comparisons with previous research

Our findings suggest that practitioners and patients perceived that engagement in self-management among patients with multimorbidity is contingent on the three inter-linked factors of capacity, responsibility and motivation. The availability and expression of these factors was moderated by the presence of high or low deprivation and/or the presence of mental and physical multimorbidity.

Structural capacity explained how patients’ ability to engage in self-management practices was partly based on their access to social, economic, and material resources. Physical and emotional capacity were also key ingredients in enabling patients to carry out multiple tasks and seize opportunities to use social resources to support pathways to self-management. Economic hardship reduced structural and emotional capacity, while interpretive capacity, in the form of know-how, increased opportunities for some patients to adopt self-management behaviours that had synergistic effects on multiple problems. As such, capacity was a necessary pre-cursor to patient engagement in self-management, but it was not sufficient.

Similarly, Shippee et al. proposed a cumulative complexity model to describe how clinical and social factors accumulate over time and interact to complicate patient care, including self-care, among patients with multimorbidity [27]. In this cumulative complexity model, the ability of patients to self-manage hinges on a balance between patient ‘workload’ of demands and patient capacity to address increasingly complicated demands. As with our findings, patient capacity here refers to the availability of financial and social resources as well as health literacy, but also includes level of physical functioning. Workload is used to denote the extent to which patients expend time and energy on health care and self-management, and on coping with life in general. In this sense Shippee et al usefully distinguish between resources that make up capacity and the use of resources that might drain capacity, further illuminating the notion that patient capacity has dynamic properties and can vary between and within patients depending on individual characteristics and circumstances. Our findings add to this model by building in an understanding that the level of certain forms of capacity is partly dependent on patient motivation. However, neither our model or that of Shippee et al has been subject to formal empirical testing yet.

Practitioners offered qualified support for the notion that all patients with multimorbidity, regardless of level of symptom or illness burden, should share responsibility to self-manage, but only if patients had the interpretative capacity to weigh up the risks and benefits of self-management practices. There is strong evidence that lower levels of health literacy are associated with poorer health status, but the notion that health literacy influences self-management through motivational and volitional determinants is contested [28]. Our practitioner data might support the argument that health literacy precedes responsibility for adopting self-management practices, but this relationship would need to be confirmed in a mediation-moderation model [29].

Patients tended to agree with practitioners’ narratives that they shared some responsibility for self-managing their illnesses. Patients’ narratives about responsibility to self-mange did however deviate to some extent along deprivation lines. Patients living in the most deprived areas tended to hold their doctor as responsible for managing and monitoring their health, whereas patients living in the least deprived areas favoured the pursuit of more individually oriented approaches to living with and managing their illnesses. This is not to assume however that patients who had greater aspirations to self-manage necessarily elevated individual over social responsibility. We have previously shown in a separate analysis that attitudes to self-management among this sample of patients did not vary between frequent or non-frequent attenders in primary care, or that self-management was more likely to be adopted by particular types of patients with specific combinations of illnesses [30]. This is in keeping with the previous findings of Townsend et al who suggested that use of healthcare among people with multimorbidity is underscored by individual characteristics of patients and their social conditions rather than specific forms of morbidity [31]. Hurd and Clarke make the compelling argument that patients’ engagement in self-management is in part driven by a desire to regain control over their bodies [32], which appeals to the notion that chronic illness, and indeed multimorbidity, present opportunities for patients to invoke a moral discourse and reinstate agency and self-determination through self-management [33].

Sinnott et al found that opportunities to engage patients with multimorbidity in patient centred approaches founded on shared decision making were limited in patients who lacked motivation [34]. Likewise we found that even where patients had capacity and responsibility, full engagement in self-management was unlikely to be successful in the absence of self-motivation. Expectations and health beliefs figured prominently in the way patients articulated motivation to engage in the full array of self-management practices, especially supported forms of self-management. Attendance at support groups was low among patients with multimorbidity because most patients did not expect that such support groups could positively transform their health or their ability to self-care. Socio-economic deprivation also shaped patients’ motivation. Patients from deprived areas were often socialised to perceive that their poor health was the norm, which limited their ambitions to improve health by self-management. By contrast patients from less deprived areas had greater motivation to self-manage because they had more opportunities to model the healthy lifestyle behaviours of peers and family. Finally, both patients and practitioners agreed that motivation was prone to being negatively affected by the presence of depression, which in turn could reduce responsibility and emotional capacity to self-manage.

The finding that *motivation* to engage in self-management may be reduced in the presence of socioeconomic deprivation is supported by a US study by Clark et al which found that patients with multimorbidity from deprived areas had lower expectations for health and successful ageing than patients from more affluent areas [35]. The concept of ‘successful ageing’ is premised on the idea that health promotion and disease prevention, principally through the adoption of health protective behaviours, can delay the onset of the harmful effects of ageing [36]. As with the patients in our study, Clark et al found that socio-economically vulnerable patients tended to focus on keeping within self-imposed limits, avoided “doing too much” and aimed to achieve basic goals such as rest and alleviation of pain rather than investing time and energy in more complex lifestyle changes. By contrast, patients with private health insurance shared “life goals” such as seeing their grandchildren grow up, working, socialising and enjoying their hobbies and saw health promotion as key to successful ageing. This difference in perspective can partly be explained by what is known as a future time perspective [37] which is a learned cognitive style of information processing that favours a focus on the future and is partly conditioned by socio-economic context [38]. In our sample, patients from deprived areas were socialised to see ill health as an expected outcome and focused on day-to-day existence rather than changing their behaviour to improve their health in the future. Additionally we also found that patients were less likely to engage in supported forms of self-management owing to a lack of expectation that such groups would benefit their health. This explanation maps onto the health belief model which suggests that individuals will act in a specific way if they believe or expect that they will benefit from those actions [39,40]. Or, alternatively, it might be for some patients multimorbidity is not perceived as a significant additional burden and as such they do not feel the need to seek out further support [31,41].

The fact that both our study and Clark et al found that for more deprived patients self-management revolved around taking medicines suggests that medication management is, for some patients, a central focus around which they can be responsible for their illnesses [42]. This highlights that practices broadly defined as ‘self-management’ (such as medication taking and lifestyle) can be construed by patients in very different ways.

### Strengths and limitations

A particular strength of this study was the inclusion of both patient and practitioner views as previous qualitative studies have tended to focus only on one group [43,44]. This allowed triangulation of emergent themes and the use of deviant cases to illuminate difference and similarity between patient and practitioner accounts. However, although qualitative work does not seek to make probabilistic generalisations it does strive to make logical generalisations to support the development of explanatory theory about how other comparable populations experience similar classes of phenomena [45]. Our efforts to achieve this level of generalizability may be weakened by the fact that the practitioners in our study were mainly GPs who were partly recruited using a convenience sample, potentially leading to selection bias.

We anticipated that socio-economic deprivation would be an important factor in determining patients’ and practitioner attitudes about self-management. We therefore used the IMD to measure the level of deprivation for each area where patients lived and where practitioners worked. While the IMD score can be used to identify the most and least deprived areas in England and to compare the levels of deprivation between areas it cannot identify individuals who are deprived and individuals who are not. As with other area level measures the IMD is thus possibly prone to ecological bias and it is recommended that both individual and area-based measures of socio-economic status are used to capture important differences in social and economic status [46]. When researching patients with multimorbidity collecting individual level data on home ownership may be particularly relevant as these patients are typically older and/or retired adults for whom the importance of other indicators of socio-economic status is much reduced [47].

Additionally, it is known that the burden of multimorbidity varies by ethnicity, with South Asian patients more likely to experience cardiovascular multimorbidity [48]. While self-management of risk factors for cardiovascular multimorbidity improves with increased multimorbidity there are important differences in outcome by ethnicity which remained to be explained [49]. Less is known about ethnic differences in self-management of other combinations of multimorbidity. We were however unable to assess the relevance of our findings among an ethnically diverse population. It will be important to explore whether the three factors identified in this study as important drivers of self-management practices in multimorbidity generalise to black and ethnic minority groups.

### Implications for future research and clinical practice

Despite recent interest in multimorbidity there is still a sparse evidence base on effective interventions [50]. In the UK and US the chronic disease self-management programme (CDSMP) is the dominant model that underpins educational and organisational interventions to enhance patient self-management in patients with single long term conditions. In this model the capacity and indeed moral responsibility for self-management is believed to rest with the individual patient, leading to an emphasis on psychological interventions to change behaviour by increasing self-efficacy (i.e. confidence to produce a desired outcome). As well as divorcing responsibility for health from health service providers, interventions that focus on individual capacity and responsibility for self-care have been criticised for not taking into account the complexity and plurality of experience of illness and how these experiences shape self-management practices [51]. There is preliminary evidence that the effects of the CDSMP may be at least as good in those with multimorbidity (if not better) [18], but levels of engagement remain poor, suggesting that a more socially informed model might increase engagement and demonstrate these benefits more widely.

Taking a lead from whole person [16] and network [52] perspectives, this may require a co-ordinated approach not just from primary care, but also from wider networks of local resources that can encourage patients with multimorbidity to engage in sustainable healthy behaviour change [53]. For example, signposting to existing community groups which help to reduce social isolation – optimising *capacity*; and by funding local peer-led healthy lifestyle initiatives – thus optimising *responsibility* through positive role modelling. Previous research has shown that general practitioners are reluctant to discuss self-management with patients with one long term condition for fear of alienating patients and disrupting the consultation [54]; this also equally applies to consultations with patients with multimorbidity [30]. However, training of primary care practitioners in theoretically informed brief behaviour change techniques [55], such as motivational interviewing, might enable them to optimise patient *motivation* and enhance engagement in self-management.

Key to the success of such initiatives might be to engage patients with multimorbidity in the co-design of interventions. Experience based co-design is an evidence based approach to involving service users in the development phase of intervention design to enhance their experience of treatment and care [56]. EBCD focuses on drawing out issues that are often hidden from view but are critical to patient experience. Understanding how capacity, responsibility, and motivation combine to support engagement in self-management may offer a way forward to crafting interventions that can benefit both practitioners and patients. This may be especially true in relation to understanding why some patients had greater interpretative capacity to identify opportunities to extract maximum benefit from self-management practices for all their conditions. Here, harnessing patient ‘know-how’ about optimal methods of self-management in the face of multimorbidity would potentially benefit other patients with less interpretative capacity.

Managing mental and physical multimorbidity is particularly challenging [57] and the findings from our study alluded to the idea that prioritising the treatment of depression in patients with multimorbidity might increase their motivation to self-manage. However, in the context of primary care, it is difficult for practitioners to routinely prioritise the management depression in people with long term conditions [21]. Effective management of mental and physical multimorbidity might therefore only be possible in the context of system level interventions such as collaborative care [58], but the effectiveness of these approaches have not been fully tested in multimorbid populations. Alternatively, there is growing interest in mindfulness interventions that offer patients opportunities to engage in synergistic approaches that address both mental and physical ill health [59], and further research about the effectiveness of these approaches in multimorbidity is warranted

## Conclusion

Managing multimorbidity is a challenge for both patients and primary care practitioners that is set to increase over the next decades owing to the growing prevalence of long-term conditions among ageing populations. This study found that, from the perspectives of patients and professionals, full engagement in self-management practices is only likely in the presence of three contingent factors: capacity; responsibility; and motivation. The operation of these factors is moderated by the presence of deprivation and physical and mental multimorbidity. The findings can be used to model the way in which individual patient characteristics and social conditions combine to form barriers to self-management in multimorbidity. Improving interpretive capacity may lead to greater responsibility and in turn motivation on the part of patients to self-manage multimorbidity. Health services can be linked to wider support networks to create therapeutic spaces in which patients can more readily express capacity, responsibility and motivation for self-management.

## Additional files

Additional file 1:
**OPTIMUM Interview Schedule - Patient.**
Additional file 2:
**OPTIMUM Interview Schedule - Practitioner.**

## Abbreviations

CHD: Coronary heart disease
COPD: Chronic obstructive pulmonary disease
DR: Doctor
GP: General practitioner
OA: Osteoarthritis
PN: Practice nurse
UK: United Kingdom
US: United States
