# Supplementary material for: Capacity, responsibility, and motivation: a critical qualitative evaluation of patient and practitioner views about barriers to self-management in people with multimorbidity
Source: BMC Health Serv Res. 2014 Oct 31;14:536. doi: 10.1186/s12913-014-0536-y (PMC4226873; doi:10.1186/s12913-014-0536-y)
Supplement: Additional file 1: — OPTIMUM Interview Schedule - Patient. [file 12913_2014_536_MOESM1_ESM.docx]

**OPTIMUM**

**Interview Schedule - Patient**

**Introduction**

Relevant Information concerning clinical characteristics such as diagnosed conditions, presence/absence of depressed mood collected from the survey, will be confirmed with the patient to introduce the topic.

**Understanding and defining self-care/ supported self-care**

*How does the patient define SC/supported SC, how do these differ, what is their understanding of these terms, how do they apply them*

- What do you think when we talk about self-care? How would you define it?
- How do you try to look after yourself? (try to get to talk about other forms of SC)
- Do you ever experience difficulties in managing your different conditions? Do you tend to manage your conditions all together or separately?
- What do you think when we talk about *supported* self-care? How would you define it?
- From your understanding what does this mean?
- What would you want from supported self-care? (e.g. if you could design a service yourself)
- What do you think are the potential benefits of self-care / supported self-care? Do you think there are any potential harms?
- Whose responsibility do you think it is to manage long-term conditions?

**Experiences of self-care and how it has evolved**

*Level of self-care, reliance on carers/professionals, confidence in maintaining self-care, social/emotional support, professional-patient relationship/patient-centredness*

- Who would you say takes the most active role in care for your conditions? In what way?
- What role do carers/healthcare professionals take in your care? How has this changed over time with the diagnosis of further conditions? Do you feel this has been adequate/enough?
- What role do you take in the management of your own conditions?
- Do you think you could do anything more than you do already? If so, what stops you?
- Do you think you manage all of your conditions equally well? Do you have support for all of them /or for some but not others?
- How confident are you in maintaining your own conditions? Is there anything you have difficulty with? What do you do if you are unsure of any aspect for managing your conditions?
- Do you feel you have enough social/emotional support for managing your conditions? Where do you get this support from? Ideally, where would you get it from?
- Does your doctor/nurse discuss self-management of your conditions with you? How much does your doctor/nurse include you in decisions about your care?

**Contact with health services/self-care support**

- **Do you know about local self-care support groups in your area? Or other resources such as on-line support groups?**

***YES*** *– Which ones, how referred (self/GP/other), feelings about it, how long attended*

- How did you find out about them?
- Have you been involved with any self-care support such as the Expert Patients Programme (EPP), disease specific programmes (eg diabetes/CHD), community support groups, on-line communities etc.? Which ones have you tried?
- How do you access these groups (attend meetings/phone lines/on-line)?
- Are they general or disease specific? How do they deal with the management of multiple conditions?
- Are you still attending? How long did you attend/how long have you attended?
- How do you feel about these groups? (have they helped/ in what way/would you recommend to others?) Do you think they helped you to deal with managing multiple conditions?
- Is there anything you now do differently as a result of having attended? Have they helped you balance the management of all your conditions together?
- Have they met your expectations? (Think in terms of social networks/peer support or encouragement/learning more about managing your conditions)
- Did you have any difficulties in finding out about/attending any of these groups? How did you overcome these difficulties?

***NO****- knowledge about resources, any referral by practice(GP/nurse), Why not tried= barriers (social, health, logistics), feeling ‘ready’, expectations*

- Have you ever discussed self-care support groups such as the Expert Patients Programme (EPP), disease specific programmes (eg diabetes/CHD), community support groups, on-line communities etc with your doctor/nurse?
- Have you ever thought of accessing any of these?
- If YES – do you think you will try any of these? Which ones interest you? What are your expectations of these groups/ what do you expect to get from them?
- If NO – why do you think you wont try them? What do you think is stopping you from trying them ? What are your expectations of such groups? Do you think you are ready to take part in such programmes?

**Barriers to supported self-care/programmes**

*Disablement, financial constraints/costs, low level health literacy, logistical problems, persistent depressive symptoms, balance between illness and QoL*

- What do you think stops you from getting involved in supported self-care programmes?
- Do you find it hard to access healthcare? What if anything do you think would help access?
- Do you think the programmes available are suitable for you? (multimorbid conditions/ethnicity/similar life experiences/age/gender etc)?

**Close**

*Any other issues not discussed, positive summary of the info they have given*

- Anything else you can think of that we haven’t already discussed and you feel is important in terms of your self-care/supported self-care?
