# Supplementary material for: Capacity, responsibility, and motivation: a critical qualitative evaluation of patient and practitioner views about barriers to self-management in people with multimorbidity
Source: BMC Health Serv Res. 2014 Oct 31;14:536. doi: 10.1186/s12913-014-0536-y (PMC4226873; doi:10.1186/s12913-014-0536-y)
Supplement: Additional file 2: — OPTIMUM Interview Schedule - Practitioner. [file 12913_2014_536_MOESM2_ESM.docx]

**OPTIMUM**

**Interview Schedule - Practitioner**

**Introduction**

The practice – list size, number of practitioners (roles/grades), levels of LTC/multimorbid LTC, patient demographics / level of social deprivation, how currently manage these patients

**Understanding and defining multimorbidity**

*Multimorbidity- outcome for patient health, diagnosis, role of depression/low mood, prioritising conditions, understanding of antagonism between conditions*

- How would you define multimorbidity?
- Could you give some examples of patients with multimorbidity?
- Do you think there are any specific challenges in managing these patients?
- What proportion of these patients struggle to cope with their conditions? Why don’t they cope well?

**Understanding and defining self-care/ supported self-care**

*How do they define SC/supported SC, how do these differ, what is their understanding of these terms, how do they apply them in practice*

- How would you describe patient self-care?
- What do you think the role of patients is in self-care? What does it entail?
- What is your understanding of supported self-care?
- How do you apply these in your practice
- How do you describe these to patients?

**Experiences of promoting self-care and how it has evolved**

*Promotion of self-care, active promotion, use of care plans, responses from patients, confidence and ability in this process*

- How do you feel about the idea of patient self-care
- What would you say your approach to self-care/supported self-care is like? (active, not a priority)
- How do patients generally receive advice about self-care?
- What do you think are the main barriers to patients engaging in self-care? What affect do you think multimorbidity has on barriers?

*Promotion of supported self-care, awareness of CDSMP eg EPP, active promotion, worth of such programmes, patients responses to suggestions (positive/negative, resistance)*

- What do you know about local self-care support programmes?
- How do you bring these to the awareness of patients?
- How well are these recommendations usually received? Any resistance? Any differences between patients with single or multiple conditions?
- What proportion of your patients do you think attend such programmes? Again, any differences between patients with single or multiple conditions?
- How do you value such programmes for patients?
- Do you think patients have the competency for self-care? Do you think multimorbidity affects ability to self-care? In what way?
- Do you think the programmes available meet the needs of your patients? Would you like any different/additional programmes available?
- If you could design a supported self-care programme for your patients, what would it look like?
- Do you think the programmes out there are suitable for multiple conditions? Do you feel they deal with the complexity of managing multiple conditions?

**Barriers to supported self-care programmes**

- Patients who do not access services

*Perceived/reported barriers (Disablement, financial constraints, low level health literacy, logistical problems, persistent depressive symptoms, balance between illness and QoL), suitability of specific programmes for multimorbid conditions*

- Do you follow-up suggestions to patients about attending supported self-care groups?
- For those patients who do not attend any, do you inquire why?
- What reasons have patients given you for not attending programmes? (Explore barriers)
- Do you give patients advice about overcoming barriers?
- What do you perceive as the impact of persistent depressive symptoms on attending programmes?
- Patients who do access services

*Motivations, benefit to patient/practice, impact on management of conditions/health/QoL, initial barriers- How were barriers overcome*

- How motivated do you find patients when they discuss supported self-care programmes?
- If initial barriers were discussed how were these overcome?
- What benefits do you think these programmes bring to the patient?
- What do you think the impact is on patient management of conditions/overall health/ quality of life?
- What benefits do you think these programmes bring to the practice? (any reduction in service use)

**Close –**

*Any other issues not discussed, summary of the info they have given*

- Anything else you can think of that we haven’t already discussed and you feel is important in terms of self-care/supported self-care in multimorbidity?
